# Sampling issues in quantitative analysis of dendritic spines morphology

**DOI:** 10.1186/1471-2105-13-213

**Published:** 2012-08-25

**Authors:** Błażej Ruszczycki, Zsuzsanna Szepesi, Grzegorz M Wilczynski, Monika Bijata, Katarzyna Kalita, Leszek Kaczmarek, Jakub Wlodarczyk

**Affiliations:** 1 Nencki Institute of Experimental Biology, Polish Academy of Sciences, Pasteura 3, Warszawa, Poland

**Keywords:** Dendritic spines, Monte Carlo simulations, Synaptic plasticity, Confocal microsopy

## Abstract

**Background:**

Quantitative analysis of changes in dendritic spine morphology has become an interesting issue in contemporary neuroscience. However, the diversity in dendritic spine population might seriously influence the result of measurements in which their morphology is studied. The detection of differences in spine morphology between control and test group is often compromised by the number of dendritic spines taken for analysis. In order to estimate the impact of dendritic spine diversity we performed Monte Carlo simulations examining various experimental setups and statistical approaches. The confocal images of dendritic spines from hippocampal dissociated cultures have been used to create a set of variables exploited as the simulation resources.

**Results:**

The tabulated results of simulations given in this article, provide the number of dendritic spines required for the detection of hidden morphological differences between control and test groups in terms of spine head-width, length and area. It turns out that this is the head-width among these three variables, where the changes are most easily detected. Simulation of changes occurring in a subpopulation of spines reveal the strong dependence of detectability on the statistical approach applied. The analysis based on comparison of percentage of spines in subclasses is less sensitive than the direct comparison of relevant variables describing spines morphology.

**Conclusions:**

We evaluated the sampling aspect and effect of systematic morphological variation on detecting the differences in spine morphology. The results provided here may serve as a guideline in selecting the number of samples to be studied in a planned experiment. Our simulations might be a step towards the development of a standardized method of quantitative comparison of dendritic spines morphology, in which different sources of errors are considered.

## Background

Dendritic spines are short (with the typical length up to 2-3 *μm* and up to 6-8 *μm* in case of very long filopodia) protrusions that harbor excitatory synapses. Dendritic spines are believed to play a major role in neuronal plasticity and integration through their structural reorganization [1-3]. Many physiological and pathological phenomena rely on brain plasticity, including learning and memory, epileptogenesis, drug addiction and post injury recovery. The quantitative analysis of spine morphology is therefore the essential problem. The morphology of spines is known to reflect their structure and function. Therefore, the morphology of spines is of relevance to many researchers who study the plasticity processes.

The enormous diversity of spines has been recognized since spines were first observed [4]. This diversity presents a sampling challenge whenever dendritic spines are analyzed quantitatively. If spines are compared among samples, the large variability of shapes exhibited by dendritic spines translates into significant variations of the selected populations morphology. Consequently, mean values that have been calculated for different spine populations also are highly variable. Therefore, a comparison of mean values among two (or more) sets of spines may not reveal existing systematic differences. These differences may be hidden by random variation (”buried in the noise”). Variation due to the process of selecting samples always persists, even under ideal experimental conditions. As pointed out in [5], the systematic changes may occur only in some small subpopulation of dendritic spines, which obscures them further in averaged data.

The concerns were raised that non-reproducibility or even contradictory results were reported in a set of experiments in which qualitatively similar results had been expected [6]. Such discrepancies could be possibly attributed to the problem of sampling. However, affirming whether indeed it is the problem of sampling, requires obtaining quantitative estimates, which obviously depend on the number of spines and samples that are studied, the statistical tests employed, and the shape of the distribution that describes the variable that is investigated.

Different kinds of sampling problems arise, depending on whether we compare different spine populations or if we track the time changes in live imaging of individual spines. There are several experimental situations in which one must compare images of different samples taken at specific time points. These cases include (a) comparisons of morphology of spines in transgenic versus wild-type animals, (b) models of neurodegenerative diseases, (c) studies of the influence of environmental factors, (d) the effect of pharmacological treatment, (e) characteristics of different parts of the brain or (f) different types of cells and (g) usage of electron-microscopy. We will focus on experiments in which measurements based on snapshots of different spines are analyzed.

The aim of our paper is to study the effectiveness of quantitative comparative methods in various experimental setups by means of Monte-Carlo simulation. We estimate the limitations in method sensitivity resulting from the sampling problem. Such estimates might be a guideline in selecting the number of samples in a new experiment or evaluating the sensitivity of experiments that have already been performed. It has to be stressed that there are other sources of variation present which originate in: the preparation of experimental samples, choice of the dendrite and the brain area, and the individual features of animals. Due to these factors, the estimates of method sensitivity resulting from sampling issues shall be treated as an upper (the best case) limit.

The simplest setup to compare morphology of spines comprises two groups of samples, that is, the treatment and the control. Selected subsets of spines would be assigned to each group. The simulations were performed by introducing in a controlled way, the systematic changes into the treatment group, while the spines in a control group were assigned randomly from a database that had been created previously. The morphological changes were assessed by performing the statistical tests in which the datum is the value of a certain variable, averaged over the sample. Alternatively, the distributions of variables could be compared using the Kolgomorov-Smirnov test, which could reveal changes that occur only in the subpopulation of spines. We investigated whether we can recover the differences we have previously introduced, changing the number of samples, the number of spines per sample, magnitude of introduced changes, variable studied, statistical test and its p-value. We looked both into the changes that affected the entire spine population and changes that affected only certain subpopulations. We focused both on the false negative rate and on the false positive rate. That is, we described the Type II Error. (i.e. the situation when the actual differences between populations were undetected), and the Type I Error (i.e. the situation when we conclude that there are differences between the groups while actually all spines originate from the same population). The latter case was simulated by comparing two control groups. In our analysis we focus on studies which measure the spine length, the head-width and the cross-sectional area (see Section ”Methods” for details).

Beside the parameters on which we focused our analysis, there are many other different two-dimensional and three-dimensional quantities describing the morphology of dendritic spines in confocal (and less frequently electron microscopy^a^) images that are commonly studied. These parameters describe (a) the overall sizes of the spines, (b) the details such as head size or neck length, establishing relations between the morphology and the spine structure and function, and (c) parameters which combine the spine shape with fluorescence intensity. Several algorithms dedicated to two-dimensional^b^ and three-dimensional spine segmentation in confocal stacks have been proposed [7]. However, the two-dimensional analysis is the most popular, primarily because of the insufficient resolution towards the optical axis prohibiting robust resolution of the dendritic spines [8]. The observance of spines that protrude mainly in the vertical direction is also limited by the resolution of current light microscopes [9]. The most popular quantities are spine length and width [10], major and minor axis of the spine head [11], head area [12], neck length [12,13], spine head volume [13,14], total spine volume estimated on integrated brightness [15] or combination of some quantities^c^.

## Methods

The resource used in the Monte-Carlo simulations was the database of variables that describe the morphology of 2499 dendritic spines originating from 34 cells that were used as controls in other experiments. The simulations were implemented in Python 2.5.4 using Scientific Python Library 0.7.0. In a single simulation run, two groups were created, a certain statistical test was performed, and the outcome was recorded. This procedure has been performed repeatability in order to assess the false negative and false positive rates (we performed respectively 2000 and 10000 simulation runs in each case).

### Preparation of dissociated cultures

Hippocampal dissociated cultures from P0 Wistar rats were prepared as follows: Brains were removed and hippocampi were isolated on ice in Dissociation Media DM (in mM: 81.8 *Na*_2_*SO*_4_; 30 *K*_2_*SO*_4_; 5.8 *MgCl*_2_; 0.25 *CaCl*_2_; 1 HEPES pH 7.4; 20 Glucose; 1 Kynureic Acid; 0.001% Phenol Red). Hippocampi were later incubated twice for 15 minutes at 37°*C* with 100U Papain (Worthington, NY, USA) in DM and rinsed 3 times in DM. Hippocampi were subsequently rinsed 3 times in plating media (MEM, 10% FBS, 1% Penicilin/Streptomycin). Hippocampi were triturated in plating medium until no clumps were visible. Cells were diluted 10 times in OptiMEM (Invitrogen), centrifuged for 10 minutes at room temperature, at 1000 rpm. The resulting cell pellet was suspended in plating medium. Cells were counted and plated at a density of 120,000 cells per 18 mm diameter coverslip (Assistent, Germany). The coverslips then were coated with 1 mg/ml poly-L-lysine (Sigma) and 2.5 *μg*/ml laminin (Roche). After three hours, the plating medium was exchanged for maintenance medium (Neurobasal-A w/o Phenol Red, 2% B-27 Supplement, 1% Penicilin/Streptomycin, 0.5 mM Glutamine, 12.5 *μM* Glutamate, 25 *μM* -mercaptoethanol) and cells were kept at 37°*C*, 5% *CO*_2_ in a humidified incubator for 2 weeks. All experiments were performed 14 to 19 days in vitro (DIV). Cells were transfected using Effectene (Qiagen) according to manufacturer protocol at 10 DIV with plasmid carrying RFP under *β*-actin promoter.

### Confocal imaging and image analysis

**Figure 1:**
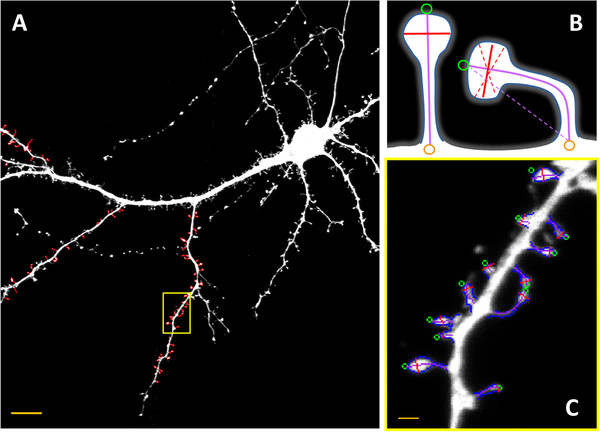
**(A) Low-power image of neuron with marked spines (red contours) selected for simulations.** Only clearly distinguishable transversally protruding spines located on the secondary dendrite were taken. Scale bar is 10*μm*. **(B)** For each spine we measured the cross sectional area, the head-width and the length. For the symmetric spine there is no ambiguity in definition of the head-width or the length. However, for the bent spines taking as the length the distance between the point at the spine tip and the foot results in underestimation of the length. Also it is not clear which distance shall represent the spine head-width (dashed red lines). For this reason we used the virtual spine skeleton to measure the length and require the head-width line to be perpendicular to this skeleton. **(C)** The magnification of a neuron with marked spines selected for simulations (Scale bar: 1*μm*). The recorded parameters were the area (blue contour), the spine length (purple curve) and the head-width (the red line)

**Figure 2:**
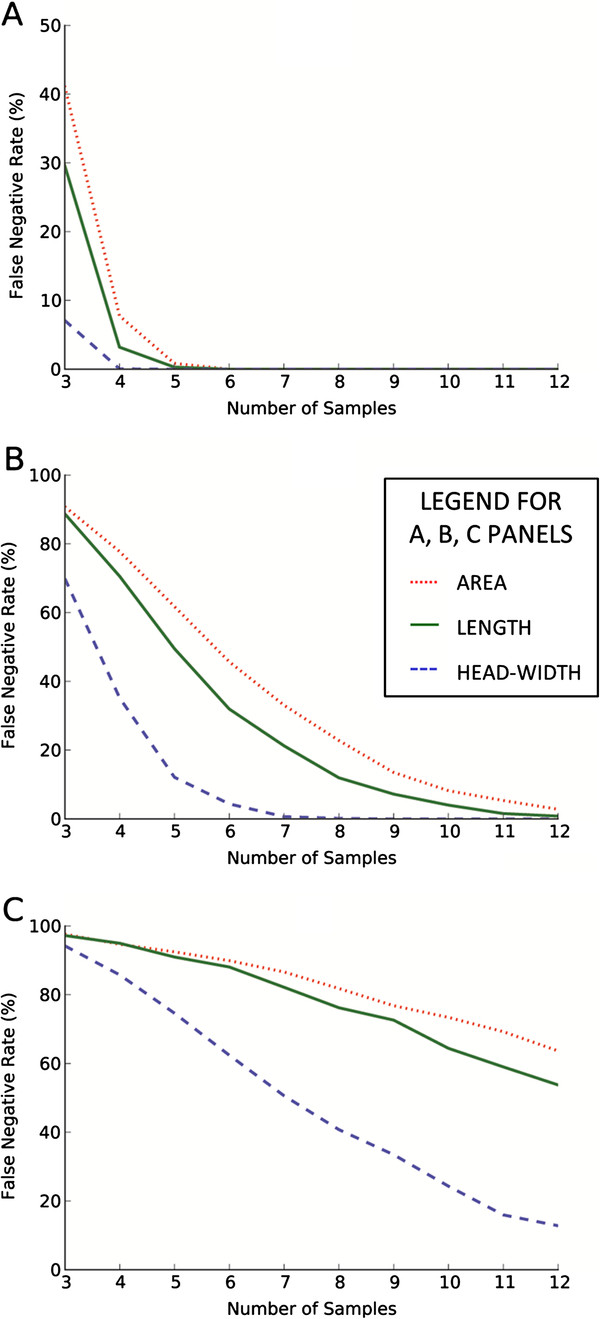
**The probabilities of committing Type II Error (concluding a “false negative”) while analyzing the results of simulated linear growth of three quantities: head-width (dashed blue line), spine area (dotted red line) and length (solid green line).** The plots correspond to linear growth by 50% **(A)**, 20% **(B)**, 10% **(C)**. The same number of samples (the x axis) was used for both the control and the treatment group, each sample contained 60 spines. The comparison of groups was based on the t-test with p-value 0.01. The ensemble of 2000 simulation runs has been used to calculate the probabilities

Images were acquired using the Leica TCS SP 5 confocal microscope with PL Apo 40 x /1.25 NA oil immersion objective using 561 nm line of diode pumped solid state laser at 10% transmission at a pixel count of 1024x1024. A series of z-stacks were acquired for a cell with step 0.4*μm*and the FWHM (full width at half the maximum) for performed observations was 0.182 *μm* while using additionally the crop function according to the Nyquist criterion resulted in the sampling density of 0.07*μm* per pixel. The images were analyzed semi-automatically using custom written software. We selected only the spines that (a) protruded in the transverse direction (in a single image plane) and (b) could be clearly distinguished. The chosen spines belonged to the secondary dendrite (see Figure 1a) The purpose of this restriction was to eliminate possible systematic differences in spine morphologies that are due to the location of spines on a dendrite with different ranks. The parameters recorded were the spine length, the head-width, the neck width, and the cross-sectional area. To determine the spine length, we measured the curvilinear length along the spine virtual skeleton (Figure 2), which was obtained by fitting the curve (the forth degree polynomial). The fitting procedure was performed by looking for a curve along which the integrated fluorescence was at a maximum. This is a certain improvement of the common definition of the spine length. The spines were distinctly bent. Therefore, the distance along a straight line between the spine tip and the base of the spine could underestimate the length of the spine (see violet dashed line in Figure 1b, right spine). The head-width was defined as the diameter of the largest spine section that was perpendicular to the virtual skeleton while the bottom part of the spine (1/3 of the spine length adjacent to the dendrite) was excluded. The neck width was defined as the thinnest spine section between the position were the head-width was measured and the point of harboring the spine into the dendrite. The practical realization of the above definitions is presented in Figure 1a. The measurements were performed using the custom-written software.

## Results and discussion

We have simulated an experiment with two groups (control and treatment) of *n* samples (representing animals or cells) and *m* spines in each sample. The variables describing the spines were drawn from a previously created database and assigned into sets representing the samples. Those that were classified into the treatment samples were subjected to further modification depending on the simulation details.

**Figure 3:**
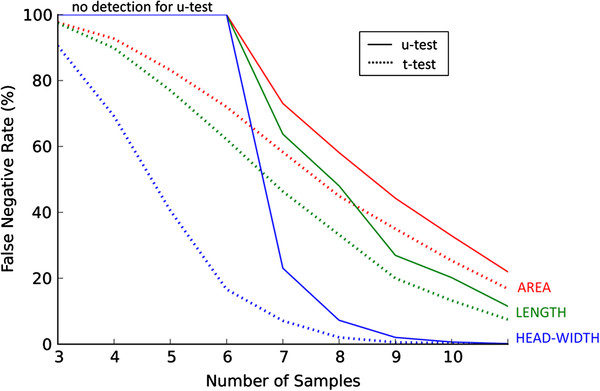
**The comparison of the effectiveness of the Mann-Whitney-Wilcoxon two-tailed u-test (solid lines) and the Student’s two-tailed t-test (dotted lines).** The linear growth of 20% has been simulated, the tests were used with p-value 0.001. The ensemble of 2000 simulations has been used with 60 spines per sample. For *n*<7 in case of the u-test there is no positive outcome. If the number of samples was small, the u-test could not provide any positive outcome due to the character of the test (the u-test is based on a discrete spectrum of order ranking obtained through permutations)

To eliminate the systematic differences in spine morphology due to the location of the spines on dendrites with different rank, special care was taken to acquire images of spines on secondary dendrites. Due to this restriction and due to the limitations resulting from the resolution of the optical microscope, we could clearly measure the morphology of roughly 30-90 spines per confocal stack (1024x1024 pixels). One of the factors contributing into the total measurement variation originates from the uncertainty of determining the spine shape. The determination of shape is restricted by the optical resolution of the microscope. In our experiment we used the RFP which gave the microscope resolution (FWHM) of 0.187 *μm*. The resolution could be improved by using a shorter excitation/emission wavelength, for example by using GFP instead of RFP it would be improved by 13% (0.024 *μm*), and while using CFP by 19% (0.034 *μm*). However, in the real experiment, the morhpometric measurements of dendritic spines are often combined with other observations, for example, with the colocalization of some proteins inside the spine, where the optical resolution is a crucial factor limiting the analysis of structures with dimensions smaller than the dimensions of a spine. Therefore, the CFP and/or GFP in such a setting would be used to visualize the additional structures. Our sampling density, established according to the Nyquist criterion, is 70 *nm* per pixel, which means an improvement of resolution by less ±1 pixel, resulting only in a slight improvement of the accuracy of measurements. As the main source of variation is the diversity of dendritic spines, the increase of resolution will not significantly influence our estimations. If the planned experiment is dedicated solely to measure the morphology of the dendritic spines, using the shortest possible wavelength might be a natural choice. However, by using longer wavelength fluorophores (especially in the case of analyzing thick samples) we have an increase of penetration depth due to smaller light scattering. If the additional structures are visualized, the assignment of the fluorophores is a matter of compromise. The typical number of spines per single scan was approximately 60. We used this number in simulations, the results of which can be found in (Figure 2) and (Figure 3). The results for other numbers of spines per sample are presented in Table 1.

**Table 1:** Minimal spine population (number of cells per group) which guarantees false negative rate below 5%

**Modeled change**	**t-test p-value**	**Parameter studied**	**Required number of cells per group**		
**in spines variable**			**(with 15, 30, 60 spines/cell)**		
10%	0.001	area	166	84	43
		length	137	70	35
		head-width	66	35	19
20%	0.001	area	47	24	14
		length	39	21	13
		head-width	21	12	8
50%	0.001	area	13	8	6
		length	11	7	5
		head-width	7	5	4
10%	0.01	area	119	62	31
		length	101	51	27
		head-width	49	26	14
20%	0.01	area	33	19	10
		length	29	15	8
		head-width	15	9	6
50%	0.01	area	9	6	4
		length	8	5	4
		head-width	5	4	3

Simulation results with various numbers of cells, spines per cell, p-values of the test, strength of systematic change and parameters studied were used to set the limits on number of analyzed cells per group in order to guarantee that the false negative rate falls below 5%. The systematic changes were modeled by the linear growth. The test groups were compared by Student’s two-tailed t-test.

In the first stage, we considered a theoretical situation in which the values of each morphometric variable under investigation grew linearly (by 10%, 20% and 50%) for any spine in the treatment group. Spine length, head-width and cross-sectional area were considered independently. The groups were compared using Students t-test. The computed false negative rate is presented in Figure 2 as a function of sample numbers.

**Figure 4:**
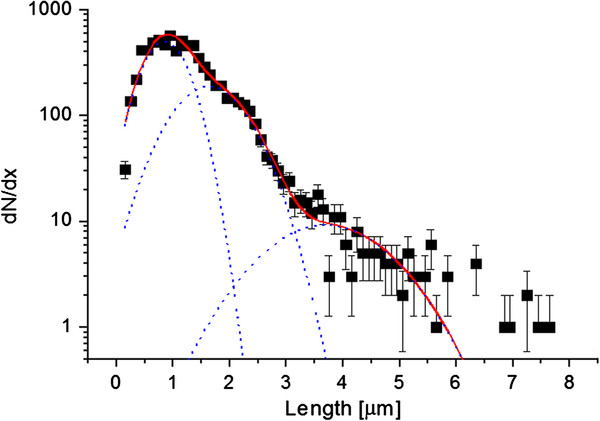
**The distribution of spine length has a highly non-Gaussian character which manifests in the large kurtosis (7.97 for the distribution studied versus 2.02 for the distribution of the spine head-width and 0 for the Gaussian distribution).** We parametrized the length distributions as a superposition of three Gaussian functions that might represent different classes of spines, yet there is still a clear deviation between the curve and points to which it was fitted for lengths >5*μm*, these points represent very long filopodia

From (Figure 2) we observe that the smallest false negative rate is obtained for head-width rather than for length or spine area. This presumably stems from the fact that the diversity in the spines population is expressed much more strongly in the area or the length distributions rather than in the one describing the head-width. In other words, the distributions of area and length deviate more from the Gaussian function because the distributions are heavy-tailed.^d^ The spine length (the variable for which kurtosis is largest) is an important variable reflecting the difference between long filopodia and short stubby spines. The distribution of spine length is given in (Figure 4).

The distribution could be modestly parametrized by a superposition of three Gaussian functions with the exception of points in the tail which represented very long filopodia (i.e., the length of filopodia was greater than 5*μm*). The presence of long filopodia might be partly responsible for the high values of false negative rates in the length analysis.

The simulation results for other settings (p-values, number of spines per sample, etc.) in a form of minimal number of samples that have to be analyzed in order to push the false negative rate below 5% are shown in Table 1.

In Figure 3 we compare the double-sided Wilcoxon-Mann-Whitney test (u-test) with the t-test, and observe that the t-test was slightly more sensitive. We observed the same behavior for other settings from Table 1. The relationship between the performance of the t-test and the performance of the u-test is not straightforward [16] and may have differed in various setups.

### Changes in Spines Subpopulations

Due to the fact that the spines exhibit a vast variation in their morphology and the biochemical composition, a model in which every spine in the treatement group is enlarged may be unrealistic. Realistically, only a certain subpopulation of spines could have been affected, or the size of changes may depend on the morphological characteristics of the spine. We will discuss here two models of spine maturation where the changes occur only in a subset of spines. We determine whether these changes can be seen in averaged data. The first one mimicked the situation in which more mature spines appeared at the cost of filopodia, and therefore there was a shift in the spectrum of the spines. Thus, we illustrated the hypothesis in which the filopodia were the precursors of dendritic spines [17].

Two distinct dynamic processes [5,18] could be present. The mature spines could have been formed directly from transient filopodia [19,20] or the filopodia could vanish without being converted directly into the mature spines as reported in [21,22]. Although these phenomena could have had very different biological origins and functionality, both could have led ultimately to similar changes in the spine spectrum. The second type of simulation targeted the idea that the small spines were more plastic and were more dependent on activity [13,23-25]. Therefore, we might have expected that the systematic changes occurred primarily in their morphology while large spines performed the spontaneous intrinsic fluctuation. As we studied the data that represented only the snapshots, we did not distinguish the underlying dynamics of the changes (e.g. whether in the treatment group, the small spines disappeared faster, or they grew into the large spines).

There are innumerable ways in which the changes could have affected certain spine subpopulations. Such changes result in modifications of the spine spectrum. We analyzed only the exemplary cases as an illustration of arising qualitative features. As an example of spine maturation, we have simulated the case in which the control group (measuring the spine lengths) was compared to the test group in which there was a 50% probability of elimination of spines with a length greater than 2*μm* In both groups we kept the same number of spines per sample.

**Figure 5:**
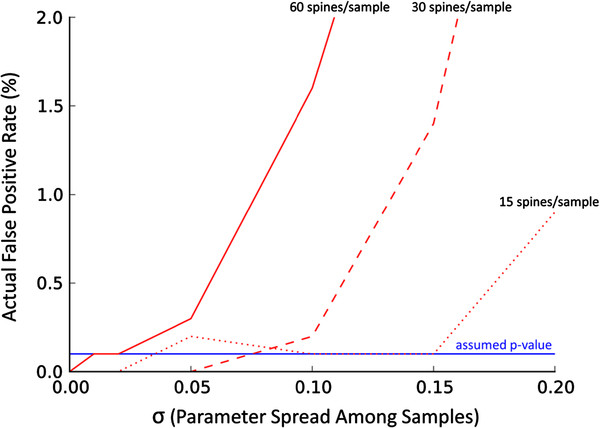
**False positive rates (probabilities of committing Type I Error) while direct comparison of two distinct population of spines is performed by means of Kolgomorov-Smirnov double sided test.** The variations of the mean values (per sample) are resulting not only from the sampling diversity but also from systematic factors (for each studied sample) affecting the measured parameter, see Results for details. The ensemble of 10000 simulation runs has been used to calculate the probabilities. The p-value was set at the level of 0.001 (the blue solid line). We have used *n*=4 animals and *m*=15,30,60 spines per sample (dotted, dashed and solid line) respectively and the spine area was used as the measured parameter. Note the superficially counter intuitive behavior: The more spines per sample we have the larger influence of systematic factors on statistics performance is observed

The general difference betweeen the simulated models of changes in spines subpopulations (maturation of spines at the cost of filopodia versus the growth of small spines) is shown in (Figure 5). In the first model the changes could be more easily detected using the t-test rather than the Kolgomorov-Smirnov test. For example, the t-test false negative rate was 5% and the K-S test false negative rate was 10% (with settings: 60 spines per sample, 30 samples and p value 0.01). In the second model the Kolgomorov-Smirnov test was significantly more sensitive than the t-test. For example, the false negative rate for the K-S test was 0.5% where as the false negative rate for the t-test was 35% (with settings: 60 spines per sample, 5 samples, p value 0.01, and 50% growth of the subpopultion of small spines defined as those with an area less than <0.8^*μm*2^). Similar behaviour was observed with other settings.

### False positive results

In order to analyse false positive results two control groups were created in an identical way and subsequently compared. In contrast to the previously discussed false negative rate, which depended on many factors, the false positive rate is determined by the level of the test significance (i.e., the p-value). We evaluated whether the test p-value coincided with actual false positive rate. In some cases these values do not coincide, for example, the t-test requires satisfying certain conditions such as distribution normality and variance homogeneity. For the parameters shown in Table 1, the p-value of the t-test agreed well with the false positive rate.

However, if we use the Kolgomorov-Smirnov test we could have discrepancy between the actual false positive rate and the p-value of the conducted test. This situation can occur, because the null hypothesis of the Kolgomorov-Smirnov test is based on the assumption that the spines were drawn from the same distribution, but it does not include any of the systematic errors that influenced the morphology of the animals, cells, or dendrites that were included in the study. Systematic errors might have originated from various factors including: (a) differences in the preparation of samples, (b) individual features of the animals or cells that were selected, and (c) differences in spine morphology due to the distance from soma, etc.

An important feature of the Kolgomorov-Smirnov test is that it does not take into account from which sample within the group a particular spine originates. Therefore, the positive outcome of the K-S test means that the two populations of spines were unlikely to have originated from the same distribution, but this does not mean that the positive outcome was caused by some systematic influence on spines in the treatment group. The difference between populations could have been caused by abnormalities in the spine morphology even in one animal.

**Figure 6:**
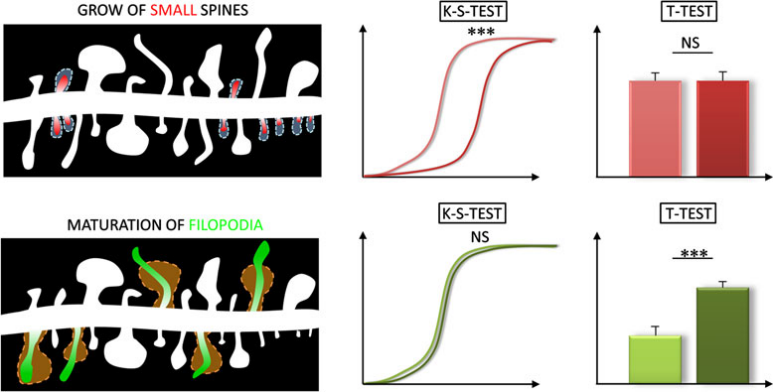
**The detectability of changes in the spine subpopulations may strongly depend on the statistical used.** For example, the growth of many small spines is much easier detected by using the K-S test rather then the t-test. In case of maturation of filopodia the situation was opposite

In our simulations we analyzed the outcomes of the Kolgomorov-Smirnov test. The parameters for each spine were subjected to additional systematic perturbations pertaining to a specific sample. Such perturbations were modeled by drawing for each sample a factor from a Gaussian distribution with the expectation value 1.0, while the variance *σ* was the controlled parameter. The measurements of every spine in a given sample were linearly modified by *σ*, and the results of the simulations, i.e., the false positive rate, were evaluated as a function of *σ*, see (Figure 6). The false positive rate increased rapidly when sigma exceeded a certain critical value somewhere between 5% and 15%. Counter to what one would expect, the critical *σ* value was smaller (i.e., we were more likely to commit Type I Error) when the samples contained more spines (while the number of samples per group and the p-value where unchanged). This counterintuitive behavior stems from the fact that it is less likely that, under the null hypothesis, we observe a given difference between the distributions when the number of spines per sample is increased. Beyond some limit, the differences between the distributions due to sampling became smaller than the other systematic effects which are not reflected in the test.

### Comparison of number of Spines in Subclasses

Another way of detecting the changes in dendritic spine morphology is to divide the studied population into discrete subclasses, and to compare the percentage of spines in each subclass within the test groups. There is no standard classification, and different researchers may use different criteria. Division of spines into subclasses may be based on the absolute criterion (i.e., taking into account whether some spine variable is below or above a certain threshold) or on the relative criterion (i.e., taking into account the ratio of two spine variables). One of the popular criteria is to divide the population into thin, mushroom, stubby spines and filopodia [26-28]. The division is based on the spine length (L), the head-width (H) and the neck-width (N). The filopodia are defined by *L*>4*μm*, stubby spines as those for which *L*/*N*>2 and the remaining spines are classified as mushroom when *H*/*N*>1.3 or thin when *H*/*N*<1.3. An example of the changes in spine population is the swallowing of spines which was modeled by an increase of the head-width with no changes in other variables. In this case we can compare the method of assessing the differences by studying the fractions of spines in subclasses, with the method which detects the differences by direct comparison of relevant spine variables in both test groups. We counted the fraction of mushroom spines or thin spines for each cell. Our simulations showed that the number of cells required to detect the simulated differences is much larger (a factor greater than 2) than the number of cells required to detect the differences by direct comparison of the head-width in both groups. We observed the same phenomenon when changing the number of spines per cell (15-120), the factor by which the spine heads grow (1.1-1.5) and the t-test p-value. It has to be noted that the comparison of the fraction of spines in subclasses fails to detect changes which occur only in a certain sub populations which do not lead to classification change of affected spines.

**Figure 7:**
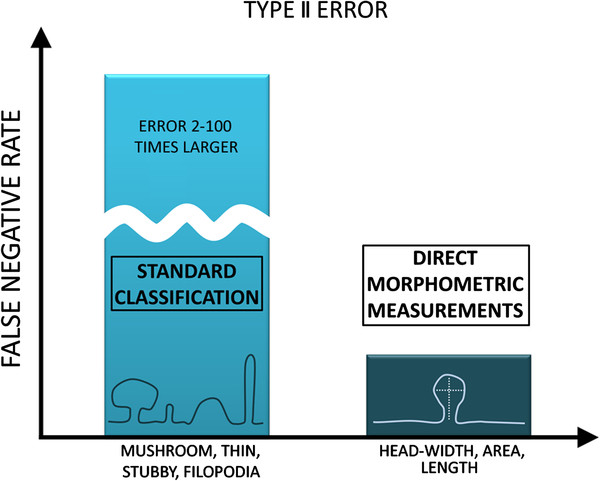
The direct morphometric measurements are much more sensitive (lower false negative rate) that the measurements based on the comparison of number of spines in subclasses.

Another possibility of division of spines into subclasses is to threshold some spine variable and to create only two subclasses. In this setting, the sensitivity of the test was studied in the following model: The spines were classified as ”large” or ”small” depending on their cross-sectional area. The threshold value was 0.65*μm*, which corresponded to the median of the distribution of the cross-sectional area. We compared the fraction of spines in subclasses for each sample in both test groups. We calculated the value of the false negative rate (by comparing the fraction of ”large spines” in the test groups) and compared it with the false negative rate of the reference test (which measured directly the cross-sectional area of the spines). We used different experimental settings such as those presented in Table 1. In any case we observed a substantially larger false negative rate (we were less likely to detect the changes) than the value obtained from the reference test, this situation is depicted in Figure 7. This value was 2-100 times larger depending on the setting we tested. We excluded those settings for which the changes were either almost always detectable by the reference test (false negative rate below 1%) or poorly detectable (false negative rate above 50%).

## Conclusions

We have described several diverse issues in quantitative analysis of spines that could interfere with the final conclusions that are drawn from a study. The results of simulations set the minimal number of samples and spines that have to be analyzed in order to achieve the assumed false negative rate. The tabularized results might be helpful in optimization of the experimental setup. Specifically, we have observed that: 

The simulation results show that systematic changes with the same magnitude would be detected more easily when the head-width, rather than the spine length or the cross-sectional area, is studied. Indeed, most of the positive results that were reported concern the changes in head-width. This also could be caused partially by the fact that head-width is a variable that is studied by most researchers interested in the morphology of dendritic spines due to: (a) known correlations that link it to the postsynaptic density, and (b) spine stability or spine head enlargement after various forms of stimulations [15,19,21-24].

For the large changes (i.e., changes greater than 50%) in any of the parameters that were studied, the differences between the groups can be easily detected because the false negative rate quickly decays (roughly exponential) as a function of the number of animals or samples. However, for more subtle changes (i.e., changes less than 10%) the decay is very slow (Figure 2c), and therefore the number of samples needed to achieve a reasonable rate of false negatives might be extremely large. In many experiments such differences might have often gone undetected. The diversity in dendritic spine population puts a practical limit on the sensitivity of the method.

In the situations that we have studied, the t-test was slightly more sensitive than the u-test (when the datum is the average value over the spines belonging to a specific animal or sample). It has to be noted here that the results of the Wilcoxon-Mann-Whitney test differed among different software packages as was reported in [29]. Presumably, this discrepancy is due primarily to the fact that the software packages use various approximations that are not documented in their manuals. For that reason, in our computations, we used exact null distribution [30].

Studies of changes that occur in the spine populations reveal that two different situations may exist: (1) The changes that effect the filopodia, which lie in the tail of the distribution of the spine length, are more easily found by comparing the mean values, rather than comparing the distributions. (2) In contrast, the changes that occur in small spines are more easily found by comparing the distributions. These changes would be “buried in the noise” if the average values alone are evaluated. These examples might represent two different general cases: (1) changes that take place in a small number of spines for which the values of some parameter describing them is large, and (2) changes in numerous population for which the measured values that contribute to the mean are small.

Detecting differences between groups by means of the Kolmogorov-Smirnov test (or any other test which compares only the shapes of distributions) could lead to underestimation of false positive rate and the false conclusion that there are significant differences between the groups whilst actually there are none (i.e., Type I Error). This situation is due to the existence of contributions (specific to cells, samples or animals) into the variations of morphological parameters. These contributions do not originate from the sampling process. The magnitude of these contributions, which is difficult to estimate, may depend on several experimental factors. The simulation results show that if this magnitude exceeds a certain value, the actual false positive rate is much higher than the assumed p-value. This problem concerns the changes in subpopulations which may be detected using a test that probes the distributions, but might not be detected by a comparison of mean values for each animal. In these cases, there should be an additional confirmation of the claimed result. One possible confirmation could be obtained by studying a fraction of spines in the subpopulation (indicated by a comparison of distributions) for each animal.

It has been customary to classify spines into subpopulations such as stubby, mushroom, thin, and filopodia. However, whether there is an actual distinction between subpopulations or we observe a continuum of shapes [18] remains an open question. The question is difficult to answer for two reasons. First, we need to define a precise criterion stating whether there are subclasses in the spine population. The fact that a distribution of length can be parameterized by three well separated Gaussian functions might be an indication that the subclasses indeed exist. The second difficulty is that the answer may depend on the type and the age of cell that are studied. The presence of a large fraction of transient, high motility spines may obscure any distinction between the classes. It would be of interest to evaluate the dependence of the distribution of mophometric parameters on cell age and type of experiment (e.g., in vivo, dissociated cultures) to see how the presence of transient spines modifies the distribution shape. Our simulations have shown that direct comparison of the variables describing dendritic spines morphology is more sensitive than the comparison of the fraction of spines in subclasses.

Starting from 1995 (especially in the studies of long term potentiation) [31] the live imaging of individual spines gained popularity. This enabled researchers to eliminate the aforementioned source of variation, as well the possible bias resulting from variations in sample preparation procedures. In this technique, an analogous sampling problem remains. The systematic changes may occur only in a certain subpopulation. Moreover, dendritic spines perform variations in their shape over time, which under certain circumstances may play an analogous role to variations in spine morphology resulting from studying different samples. Whether the dynamic spine fluctuations are sufficiently significant to possibly overshadow the systematic changes depends on the details of the experiment. The reported value of such fluctuations was approximately ∼5*μm*/100*s*for young cells in dissociated cultures, but it dropped to approximately ∼0.3*μm*/100*s* in cultures that were 20 days old [32]. In these cases, the measured value represents the largest absolute disparity between two spine contours. Because some experiments measure the impact of direct spine stimulations in a short interval (minutes or tens of minutes) the effects of motility can be ignored. However, they can become significant in experiments that measure more subtle relations, for example the correlation between environmental enrichment and changes in spine morphology over a period of several days.

Although there is no straightforward prescription for the optimal method and size of the spines population to be analyzed, special attention should be taken to understand the origin and to estimate false negative and false positive rates in the performed statistics. Misapplication may lead to a high rate of non-repeatability and to drawing frivolous conclusions from the experiment.

## Endnotes

^a^The comparative analysis of the morphology of dendritic spines has been developed starting from the quantification of the electron-microscopy section images. The shape parameter that is usually taken into consideration is the cross-sectional area. In the single section estimates, a number of spines cut by a subjective cross-section or projection was observed. The measured quantities depend significantly on the way the section cut the spine, which introduced a large uncontrollable source of variation. When high-resolution optical microscopes and three-dimensional reconstruction of serial-sectioning electron microscopy (SSEM) were introduced it became possible to use these techniques to quantify the parameters.The three-dimensional reconstruction of SSEM images is very labor-intensive [27]. Therefore this technique has the disadvantage that the resulting number of analyzed samples and spines per sample is very small.^b^There was a study [31] which corrected the 2-dim distribution of the spine length in order to reconstruct the three-dimensional distribution. The technique that was employed was convoluting the distribution with the function representing inverse stereological projection. It was based on the obvious assumption that the protrusion angles of observed spines were randomly distributed, which in practice however requires, that projections of all spines, including those protruding along the z-axis, are measured. The projections of these spines cannot be clearly measured as they are overshadowed by the dendrite.^c^There was a study [33] aimed at constructing the metric distances of dendritic spines using mathematical tools such as principal component analysis or large deformation diffeomorphic metric mapping. These techniques permit the isolation of a most comprehensive set of uncorrelated parameters that describe the shape of the spines. However, this approach does not establish the connection between the constructed metrics and the structure/functionality quantifications. The biological applications of such methods in the discussed domain are currently unknown.^d^Kurtosis is one of the measures that shows how a given distribution differs from the Gaussian function. Kurtosis is based on the fourth moment of the population and vanishes for the Gaussian function. The largest values of kurtosis were found for spine-length (7.96) and for area (7.38). For spine head-width, kurtosis had a much smaller value (2.02). These observations do not exactly coincide with the observed fact that the changes in the spine area are the most difficult to detect. However, other details of the distribution such as higher moments, could also be important.

## Competing interests

The authors declare that they have no competing interests.

## Authors’ contributions

BR and JW conceived of the study. BR developed the algorithms and performed the simulations. JW designed the imaging experiment. ZS and MB performed the experiments and analyzed the data, KK contributed the data. BR, GW, LK and JW wrote the original manuscript. GW and LK commented on the manuscript with important intellectual contributions. All authors read and approved the final manuscript.
